# Correction: Frequency and distribution of corneal astigmatism and keratometry features in adult life: Methodology and findings of the UK Biobank study

**DOI:** 10.1371/journal.pone.0229866

**Published:** 2020-02-27

**Authors:** The PLOS ONE Staff

## Notice of republication

This article was republished on February 12, 2020, to correct errors in the Introduction and author byline. The publisher apologizes for these errors. Please download this article again to view the correct version. The originally published, uncorrected article and the republished, corrected articles are provided here for reference.

## Supporting information

S1 File Originally published, uncorrected article. (PDF)

S2 File Republished, corrected article. (PDF)

## References

[1] Pontikos N, Chua S, Foster PJ, Tuft SJ, Day AC, UK Biobank Eye and Vision Consortium (2019) Frequency and distribution of corneal astigmatism and keratometry features in adult life: Methodology and findings of the UK Biobank study. PLoS ONE 14(9): e0218144 10.1371/journal.pone.0218144 31536508 PMC6752876

