# Supplementary material for: Frequency and distribution of corneal astigmatism and keratometry features in adult life: Methodology and findings of the UK Biobank study
Source: PLoS One. 2019 Sep 19;14(9):e0218144. doi: 10.1371/journal.pone.0218144 (PMC6752876; doi:10.1371/journal.pone.0218144)
Supplement: S1 File — (DOCX) [file pone.0218144.s001.docx]

Frequency and Distribution of Corneal Astigmatism and Keratometry Features in Adult Life: Methodology and Findings of the UK Biobank Study

**Supplementary Materials**

**Figure A. Asymmetry of keratometry measures in the UKBB**. a) Measured corneal astigmatism and cylindrical power in left eye is on average higher than in right eye as per the line of best-fit (red). This has been previously reported by Cumberland et al 2015 [1]. b) Corneal power and spherical power are symmetric in both eyes.

**Figure B. Distribution of corneal astigmatism.** (a) Distribution of corneal astigmatism in right (red) and left (blue) eyes. (b) Logarithmic scaling of corneal astigmatism renders the distribution more symmetric.

**Figure C. Age in relation to cylindrical power and corneal power.** (A and B) Age vs cylindrical power: Cylindrical power increases significantly with age both in right (B=0.011 (0.010 to 0.011), P<0.001) and left eye (B=0.010 (0.009 to 0.010), P<0.001). (C and D) Age vs corneal power: Corneal power in right eye increases significantly with age (B=0.015 (0.014 to 0.016), P<.001) and in left eye (B=0.015 (0.014 to 0.016), P<.001). All P values estimated from linear regression (t statistic).

**Figure D. Eye conditions in relation to corneal astigmatism and full-time education completion age.** (a) Distribution of log-transformed corneal astigmatism in right eye for eye conditions affecting the right eye and in left eye. Strabismus, amblyopia and astigmatism yield the highest values of cornea astigmatism while presbyopia yields the lowest. (b) Logistic regression of right and left corneal astigmatism against amblyopia and strabismus shows strong significant positive association, OR=1.98 and OR=1.73 respectively. (c) Distribution of age completed full-time education and eye condition. Individuals with self-reported astigmatism appear to leave full-time education later than other eye conditions.

**Figure E. Interaction between age, gender and skin colour with corneal astigmatism.** Brown skin have lower corneal astigmatism than very fair skin.


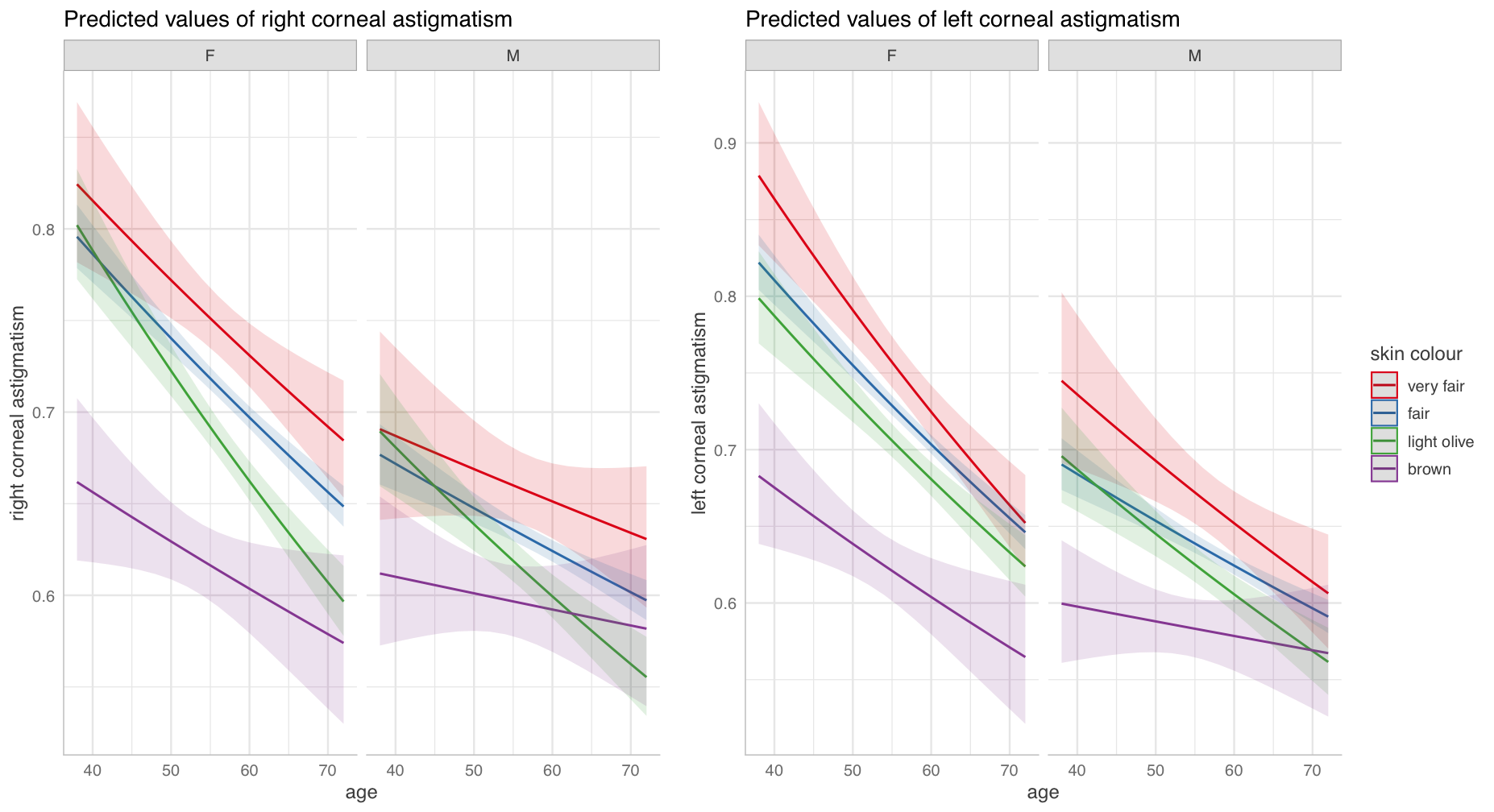


**Figure F. Three-way interaction between alcohol-intake, age and gender with corneal astigmatism.** “never-drinkers” and “daily drinkers” showing a clear interaction effect with gender.


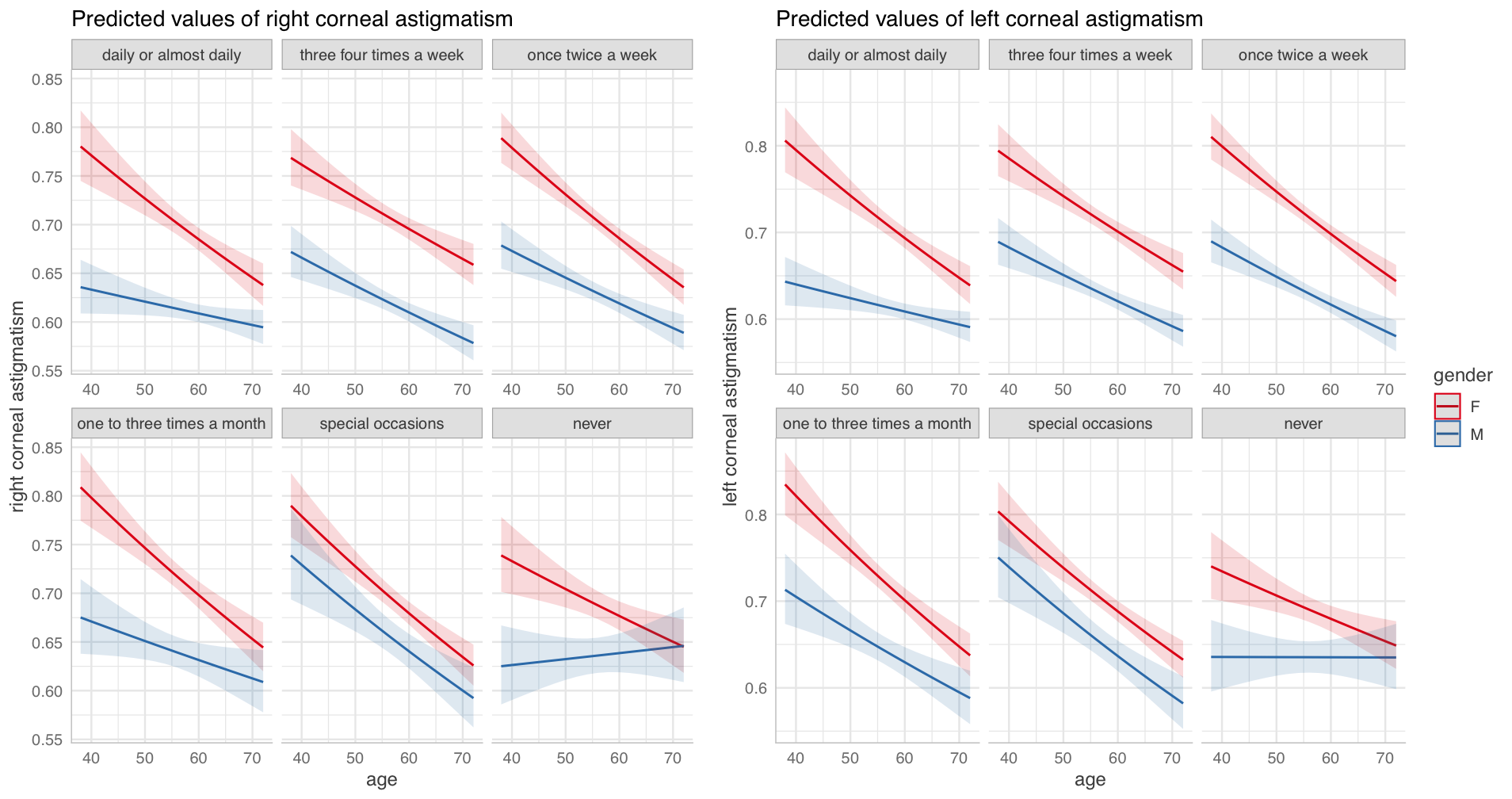


**Figure G. Classes of astigmatism in relation to age**. Prevalence of the three different classes of axis of astigmatism across age groups in the UKBB (A and B). Mean corneal astigmatism per axis of astigmatism class across age groups (C and D). ATR=against-the-rule, OB=oblique and WTR=with-the-rule.

**Figure H. Association between age and Townsend deprivation index.** There is a significant negative association between age and Townsend deprivation index, with older individuals tending to live in less deprived areas (B=-0.05 (-0.05 to -0.04), P<0.001).

**Table A. Recoding of self-reported variables from UKBB touch screen and their recoding in the statistical analysis.**

| **Question** | **Answers** | **Coding** | **Recoding** |
| --- | --- | --- | --- |
| **Smoking status (data-field: 1239)**  **“Do you smoke tobacco now?** | Yes, on most or all days | 1 | 2 |
|  | Only occasionally | 2 | 1 |
|  | No | 0 | 0 |
|  | Prefer not to answer | -3 | NA |
| **Alcohol intake frequency (data-field: 1558)**  **“About how often do you drink alcohol?”** | Daily or almost daily | 1 | 5 |
|  | Three or four times a week | 2 | 4 |
|  | Once or twice a week | 3 | 3 |
|  | One to three times a month | 4 | 2 |
|  | Special occasions only | 5 | 1 |
|  | Never | 6 | 0 |
|  | Prefer not to answer | -3 | NA |
| **Skin colour (data-field: 1717)**  **"What best describes the colour of your skin without tanning?"** | Very fair | 1 | 1 |
|  | Fair | 2 | 2 |
|  | Light olive | 3 | 3 |
|  | Dark olive | 4 | 4 |
|  | Brown | 5 | 5 |
|  | Black | 6 | 6 |
|  | Do not know | -1 | NA |
|  | Prefer not to answer | -3 | NA |
| **Use of sun/UV protection (data-field: 2267)**  **"Do you wear sun protection (e.g. sunscreen lotion, hat) when you spend time outdoors in the summer?"** | Never/rarely | 1 | 1 |
|  | Sometimes | 2 | 2 |
|  | Most of the time | 3 | 3 |
|  | Always | 4 | 4 |
|  | Do not go out in sunshine | 5 | 0 |
|  | Do not know | -1 | NA |
|  | Prefer not to answer | -3 | NA |
| **Presence of diabetes (data-field: 2443)**  **“Has a doctor ever told you that you have diabetes?”** | Yes | 1 | 1 |
|  | No | 0 | 0 |

**Table B. Results of univariable regression in 83,751 study participants in the UKBB for right and left eye log-transformed corneal astigmatism.** Significant associations are highlighted in bold. P values for B coefficients of the multivariable linear regression are derived from a t-test. 95% CI=95% confidence interval.

| Description | right eye  univariate B (95% CI) | P value | left eye  univariate B (95% CI) | P value |
| --- | --- | --- | --- | --- |
| **Age, years** | **-0.005 (-0.006 to -0.005)** | **<0.001** | **-0.006 (-0.007 to -0.005)** | **<0.001** |
| **Sex (baseline = F)** | **-0.115 (-0.125 to -0.105)** | **<0.001** | **-0.122 (-0.132 to -0.112)** | **<0.001** |
| **Ethnicity (baseline=white)**  **asian** | **-0.112 (-0.139 to -0.084)** | **<0.001** | **-0.141 (-0.169 to -0.114)** | **<0.001** |
| **black** | **-0.083 (-0.113 to -0.054)** | **<0.001** | **-0.082 (-0.111 to -0.053)** | **<0.001** |
| mixed | -0.019 (-0.072 to 0.034) | 0.489 | -0.020 (-0.073 to 0.033) | 0.455 |
| chinese | 0.004 (-0.074 to 0.082) | 0.922 | -0.028 (-0.106 to 0.050) | 0.489 |
| **Age completed full time education** | **0.004 (0.002 to 0.007)** | **<0.001** | **0.006 (0.004 to 0.009)** | **<0.001** |
| **Skin colour, lighter to darker*** | **-0.035 (-0.041 to -0.030)** | **<0.001** | **-0.035 (-0.040 to -0.030)** | **<0.001** |
| **Use of UV protection** | **0.026 (0.020 to 0.031)** | **<0.001** | **0.024 (0.019 to 0.030)** | **<0.001** |
| **Alcohol intake, never to daily**** | **-0.009 (-0.013 to -0.006)** | **<0.001** | **-0.009 (-0.012 to -0.005)** | **<0.001** |
| Season of assessment (baseline=spring)  autumn | -0.004 (-0.017 to 0.009) | 0.539 | -0.010 (-0.024 to 0.003) | 0.13 |
| winter | -0.012 (-0.025 to 0.002) | 0.086 | -0.011 (-0.025 to 0.002) | 0.102 |
| summer | 0.019 (0.005 to 0.033) | 0.009 | 0.018 (0.004 to 0.032) | 0.011 |
| **Corneal corrected IOP, mmHg** | **-0.008 (-0.010 to -0.007)** | **<0.001** | **-0.009 (-0.010 to -0.008)** | **<0.001** |
| Corneal resistance factor | -0.002 (-0.004 to -0.000) | 0.05 | 0.001 (-0.001 to 0.003) | 0.228 |
| **Corneal hysteresis** | **0.006 (0.004 to 0.008)** | **<0.001** | **0.009 (0.007 to 0.012)** | **<0.001** |
| **Height, m** | **-0.003 (-0.004 to -0.003)** | **<0.001** | **-0.003 (-0.004 to -0.003)** | **<0.001** |
| Weight, 10 kg | -0.001 (-0.001 to -0.001) | <0.001 | -0.001 (-0.001 to -0.001) | <0.001 |
| BMI, kg/m2 | 0.000 (-0.001 to 0.001) | 0.761 | -0.000 (-0.001 to 0.001) | 0.71 |
| **SBP, mmHg** | **-0.001 (-0.002 to -0.001)** | **<0.001** | **-0.001 (-0.002 to -0.001)** | **<0.001** |
| **DBP, mmHg** | **-0.001 (-0.002 to -0.001)** | **<0.001** | **-0.001 (-0.002 to -0.001)** | **<0.001** |
| Townsend deprivation index | 0.001 (-0.001 to 0.002) | 0.355 | 0.002 (0.001 to 0.004) | 0.004 |
| Smoker (baseline = 0) | -0.025 (-0.042 to -0.009) | 0.003 | -0.013 (-0.030 to 0.003) | 0.117 |
| Age Asthma Diagnosed, self reported | -0.001 (-0.002 to -0.000) | 0.004 | -0.001 (-0.002 to 0.000) | 0.08 |
| Diabetes, doctor diagnosed  (baseline = 0) | -0.047 (-0.070 to -0.025) | <0.001 | -0.034 (-0.056 to -0.011) | 0.004 |

* Skin colour is coded as: very fair=1, fair=2, light olive=3, dark olive=4, brown=5, black=6; ** Alcohol intake is coded as: never=0, special occasions=1, one to three times a month=2, once twice a week=3, three four times a week=4, daily=5.

**Table C. Results of multivariable regression in 83,751 study participants in the UKBB for right and left eye log-transformed corneal astigmatism.** Only parameters which were significant in the univariable regression were included in the multivariable regression. Significant associations are highlighted in bold. P values for B coefficients of the multivariable linear regression are derived from a t-test. 95% CI=95% confidence interval.

| Description | right eye multivariate B (95% CI) | P value | left eye multivariate B (95% CI) | P value |
| --- | --- | --- | --- | --- |
| **Age, years** | **-0.004 (-0.005 to -0.003)** | **<0.001** | **-0.004 (-0.004 to -0.003)** | **<0.001** |
| **Sex (Ref = F)** | **-0.057 (-0.073 to -0.040)** | **<0.001** | **-0.083 (-0.100 to -0.066)** | **<0.001** |
| Ethnicity (baseline=white) asian | -0.014 (-0.054 to 0.027) | 0.5 | -0.066 (-0.107 to -0.026) | 0.001 |
| black | 0.030 (-0.015 to 0.076) | 0.192 | -0.020 (-0.067 to 0.027) | 0.404 |
| mixed | -0.058 (-0.121 to 0.004) | 0.068 | -0.036 (-0.100 to 0.028) | 0.267 |
| chinese | -0.074 (-0.176 to 0.027) | 0.152 | -0.061 (-0.164 to 0.041) | 0.24 |
| **Age completed full time education** | **0.005 (0.002 to 0.007)** | **<0.001** | **0.006 (0.003 to 0.008)** | **<0.001** |
| **Skin colour , lighter to darker*** | **-0.034 (-0.043 to -0.025)** | **<0.001** | **-0.029 (-0.038 to -0.020)** | **<0.001** |
| Use of UV protection | 0.006 (-0.001 to 0.012) | 0.089 | 0.000 (-0.006 to 0.007) | 0.904 |
| Alcohol intake, never to daily** | -0.008 (-0.012 to -0.004) | <0.001 | -0.006 (-0.010 to -0.002) | 0.004 |
| **Corneal corrected IOP, mmHg** | **-0.006 (-0.008 to -0.005)** | **<0.001** | **-0.006 (-0.008 to -0.005)** | **<0.001** |
| Corneal hysteresis | -0.007 (-0.009 to -0.004) | <0.001 | -0.003 (-0.006 to -0.000) | 0.033 |
| Height, m | -0.002 (-0.003 to -0.001) | 0.001 | -0.001 (-0.002 to 0.000) | 0.117 |
| **Weight, 10 kg** | **0.001 (0.000 to 0.001)** | **<0.001** | **0.001 (0.000 to 0.001)** | **<0.001** |
| SBP, mmHg | -0.000 (-0.000 to 0.000) | 0.779 | -0.000 (-0.001 to 0.000) | 0.352 |
| DBP, mmHg | 0.001 (-0.000 to 0.001) | 0.15 | 0.000 (-0.000 to 0.001) | 0.21 |
| Townsend deprivation index | |  | 0.004 (0.002 to 0.006) | <0.001 |
| Diabetes, doctor diagnosed (baseline = 0) diabetes1 | 0.006 (-0.019 to 0.031) | 0.646 |  |  |

* Skin colour is coded as: very fair=1, fair=2, light olive=3, dark olive=4, brown=5, black=6; ** Alcohol intake is coded as: never=0, special occasions=1, one to three times a month=2, once twice a week=3, three four times a week=4, daily=5.

1. [Cumberland PM, Bao Y, Hysi PG, *et al.* Frequency and Distribution of Refractive Error in Adult Life: Methodology and Findings of the UK Biobank Study. *PLoS One* 2015;**10**:e0139780.](http://paperpile.com/b/RWzgHy/pIld)
