# Supplementary material for: Correction: Frequency and distribution of corneal astigmatism and keratometry features in adult life: Methodology and findings of the UK Biobank study
Source: PLoS One. 2020 Feb 27;15(2):e0229866. doi: 10.1371/journal.pone.0229866 (PMC7046213; doi:10.1371/journal.pone.0229866)
Supplement: S2 File — (PDF) [file pone.0229866.s002.pdf]

RESEARCH ARTICLE

Frequency and distribution of corneal astigmatism and keratometry features in adult life: Methodology and findings of the UK Biobank study

Nikolas Pontikos[1,2]*, Sharon Chua[1,2], Paul J. Foster[1,2], Stephen J. Tuft[1,2], Alexander C. Day[1,2], UK Biobank Eye and Vision Consortium¶

1 UCL Institute of Ophthalmology, London, England, United Kingdom, **2** NIHR Biomedical Research Centre, Moorfields Eye Hospital, London, England, United Kingdom

¶ Collaborators on behalf of the UK Biobank listed in the acknowledgments.
* n.pontikos@ucl.ac.uk

Check for updates

OPEN ACCESS

Citation: Pontikos N, Chua S, Foster PJ, Tuft SJ, Day AC, UK Biobank Eye and Vision Consortium (2019) Frequency and distribution of corneal astigmatism and keratometry features in adult life: Methodology and findings of the UK Biobank study. PLoS ONE 14(9): e0218144. https://doi.org/10.1371/journal.pone.0218144

Editor: Ahmed Awadein, Faculty of Medicine, Cairo University, EGYPT

Received: May 20, 2019

Accepted: August 27, 2019

Published: September 19, 2019

Copyright: © 2019 Pontikos et al. This is an open access article distributed under the terms of the Creative Commons Attribution License, which permits unrestricted use, distribution, and reproduction in any medium, provided the original author and source are credited.

Data Availability Statement: The data underlying the results presented are third-party data publicly available from the UK Biobank (https://www.ukbiobank.ac.uk/). The approved study can be found here on the UK Biobank website: https://www.ukbiobank.ac.uk/2016/05/dr-alexander-day-moorfields-eye-hospital-nhs-foundation-trust-london/. The fields which were used are described in the following GitHub code repository: https://github.com/pontikos/UKBB.

Abstract

Purpose

To describe corneal astigmatism in the UK Biobank population and to look for associations with other biometric variables and socio-demographic factors.

Methods

This analysis included a subsample of 107,452 participants of the UK Biobank study who underwent an enhanced ophthalmic examination including autorefractor keratometry (Tomey RC 5000, Tomey Corp., Nagoya, Japan). Participants were recruited from across the United Kingdom between 2006 and 2010, and all were between 40 to 69 years. After quality control and applying relevant exclusions, data on corneal astigmatism on 83,751 participants were included for analysis. Potential associations were tested through univariable regression and significant parameters carried forward for multivariable analysis.

Results

In univariable analysis, the characteristics significantly associated with higher corneal astigmatism (P<0.001), by order of magnitude were, female gender, white ethnicity, lighter skin colour, use of UV protection, lower alcohol intake, lower corneal-compensated intraocular pressure (ccIOP), older age at completion of education, younger age, higher Townsend deprivation index, lower height and lower systolic blood pressure. After inclusion in the multivariable analysis, gender, skin colour, alcohol intake, age at completion of full-time education, ccIOP, age and Townsend deprivation score remained significant (all P<0.001). Increased corneal astigmatism was also found to be significantly associated with amblyopia or strabismus.

Funding: The collection of eye & vision data in UK Biobank was supported in part by a grant from the NIHR Biomedical Research Centre at Moorfields Eye Hospital and UCL Institute of Ophthalmology. NP is funded by a Moorfields Eye Charity Career Development Award (https://www.moorfieldseyecharity.org.uk/career-development-awards) (award number R190031A). The UK Biobank Eye and Vision Consortium was supported by a grant from The Special Trustees of Moorfields Eye Hospital (now Moorfields Eye Charity). The funders had no role in study design, analysis, decision to publish, or preparation of the manuscript. The views expressed in this article are those of the authors and not necessarily those of the NHS, the NIHR or the Department of Health.

Competing interests: The authors have declared that no competing interests exist.

Conclusions

This analysis confirms previous associations with astigmatism such as younger age and female gender, and identified novel risk factors including lighter skin colour, lower alcohol intake, later age having completed full time education later, lower ccIOP and higher Townsend deprivation index. Further research is needed to investigate these novel associations.

Introduction

Uncorrected refractive error is the leading cause of moderate to severe visual impairment in all age groups globally [1,2]. Refractive error (ametropia), is a significant public health burden, frequently associated with worse visual acuity and higher risk of amblyopia. The two major components of refractive error in the eye are astigmatism and the spherical refractive error (myopia or hyperopia). Astigmatism is caused by a corneal component and lenticular component. Corneal astigmatism occurs when there are differences in the radius of curvature of the cornea in different meridians such that there is a different focal point for each meridian, with an area of intermediate focus between the two termed the conoid of Sturm[3]. The two meridians are defined as either the steep meridian (also known as the strong meridian) and the flat meridian (also known as the weak meridian). The magnitude of corneal astigmatism has been reported to vary with age [4,5] and there is a shift from the steepest corneal meridian from the vertical (with-the-rule) to the horizontal meridian (against-the-rule)[6–9]. Data on the prevalence and severity of corneal astigmatism is typically obtained from case series of patients undergoing cataract surgery [9–12], with limited data from population based cross-sectional studies or large cohort studies [13]. The UK Biobank (UKBB) study[14–16] recruited over 500,000 men and women aged 40 to 69 years between 2006–2010 from the general population. In 2009 the study protocol was updated to include measurement of ocular data including corneal keratometry, on a subset of these [16]. The aim of our analysis is to describe corneal astigmatism and derived variables in the UKBB population, to look for associations with other biometric variables, socio-demographic factors, and eye conditions.

Materials and methods

UKBB participants

The UKBB participants have previously been described in detail by Allen et al (2012) [16]. In brief, all adults aged between 40 and 69 years old who were registered with the UK National Health Service and living within 25 miles of one of the 22 participating study sites were invited to participate. From a total of 9.2 million postal invitations, 503,325 participants were recruited between 2006 and 2010 (response rate of 5.5%) and after accounting for withdrawals; data on 502,642 participants were available for analysis. All those recruited completed detailed questionnaires on their lifestyle, socioeconomic status, environment and health, and had a number of physiological measures from urine, saliva and blood samples. Further information can be found on the UK Biobank online data showcase (http://biobank.ctsu.ox.ac.uk/crystal/label.cgi).

Ethics. All UK Biobank participants gave written, informed consent. The UK Biobank study was conducted under approval from the NHS National Research Ethics Service (Ref. 11/NW/ 0382), and anonymised data were provided from UK Biobank under application reference 10536.

Eye measurements. Six of the recruiting centres performed an ophthalmic assessment [17] that included LogMAR visual acuity, autorefraction and keratometry (Tomey RC 5000 auto-ref-keratometer Tomey Corp., Nagoya, Japan), intraocular pressure (IOP) (Goldmann-correlated and Corneal-compensated) and corneal biomechanics (both Ocular Response Analyzer, Reichert, Depew, NY, USA). In total, 117,279 (23.3%) of those enrolled had an ophthalmic assessment. The Tomey RC 5000 examination produced autorefraction and keratometric measurements for each eye (http://biobank.ctsu.ox.ac.uk/crystal/label.cgi?id=100014). The Reichert Ocular Response Analyser (Reichert Corp., Philadelphia, PA), measures the biomechanical distortion of the cornea produced by a puff of air for each eye. Measurements included corneal hysteresis, corneal resistance factor and corneal-compensated intraocular pressure (ccIOP) (http://biobank.ctsu.ox.ac.uk/crystal/label.cgi?id=100015). Participants who had eye surgery within the previous 4 weeks or those with possible eye infections did not have IOP measured.

Self-reported eye conditions. The UKBB touchscreen questionnaire allowed participants to report if they had eye disorders or eye diseases, any injury or trauma, which eye was affected and when it was diagnosed. Refractive eye conditions included astigmatism, myopia, hyperopia, presbyopia, strabismus and amblyopia. Eye diseases include diabetic retinopathy, glaucoma, cataract or age-related macular degeneration.

Socio-economic status and ethnicity. The Townsend deprivation index was determined using the participant's postcodes at recruitment. The Townsend deprivation index has a UK mean of zero, with negative being less deprived and positive being more deprived. Ethnicity choices included white (English/Irish or other white), Asian or British Asian (Indian/Pakistani/Bangladeshi or other Asian), black or black British (Caribbean, African, or other black), Chinese, mixed (white and black Caribbean or African, white and Asian, or other mixed ethnicity), or other ethnic group (not defined).

Lifestyle and environment. The UKBB touchscreen questionnaire also offered questions to participants about their lifestyle, health and environment. In particular, smoking status, alcohol intake frequency, use of sun/UV protection, skin colour without tanning, presence of diabetes. The possible questions/answers and their encoding are explained in more detail in the Table A in S1 File.

Physical measures. Blood pressure and heart rate were measured using the HEM-70151T digital blood pressure monitor (Omron, Hoofddorp, The Netherlands). Weight was measured with the BV-418 MA body composition analyzer (Tanita, Arlington Heights, IL). Height was measured using a Seca 202 height measure (Seca, Birmingham, UK). Body mass index (BMI) was calculated as weight in kg divided by height in m^2. Waist circumference at the level of the umbilicus was measured using a Wessex non-stretchable tape measure.

Participant selection

Of the 502,642 participants in UK Biobank, 109,935 had 3mm steep or flat corneal meridian measurement values available for both eyes from which corneal astigmatism measurements could be derived. Participants were excluded if they had any of the following: previous laser refractive eye surgery (n = 7440), previous eye surgery (for cataract, glaucoma or corneal graft) (n = 8051), unreliable 3mm asymmetry index (n = 12,910) or an unreliable keratometry result (n = 6916). This left a total of 83,751 individuals for further analysis.

Corneal astigmatism, mean corneal power and axis of astigmatism

Corneal astigmatism was defined as the 3mm steep meridian minus the 3mm flat meridian. The average of these two values was defined as the mean corneal power. The axis of

astigmatism was defined as the angle of the steep meridian. The axis of astigmatism was categorised as with-the-rule if the angle was between 60 and 119 degrees, against-the-rule if the angle was in the intervals 0 to 29 or 150 to 180, otherwise it was categorised as oblique [18].

Statistical methods

We explored the distribution of corneal astigmatism and compared this to previously published studies. We tested the association of mean corneal power and axis of astigmatism with age in both eyes by linear regression. Univariable linear regression and multivariable linear regression statistical analysis models were applied to investigate predictors of corneal astigmatism. Non numeric independent variables were re-coded according to Table A in S1 File. P values for the B coefficients of the linear regressions were derived using a t-test. To account for multiple testing, a Bonferroni corrected P value threshold of < 0.001 was applied to avoid false-positives due to the large number of tests carried out. Only parameters that showed significant association in the univariable analysis were included in the multivariable analysis. Since we found that corneal astigmatism measurements were slightly asymmetric, with left eye having on average higher corneal astigmatism than right eye (Fig A in S1 File) as previously reported by Cumberland et al [17], we repeated statistical analysis in both eyes and only reported parameters which were consistently significantly associated in both eyes. We also repeated the statistical associations with a log-scaled corneal astigmatism variable since the P value derived from a t-test in a linear regression assumes a normally distributed response variable rather than a skewed distribution (Fig B in S1 File). All analyses were performed using R statistical software version 3.2.3. The code is available at https://github.com/pontikos/UKBB/.

Results

Participant selection and distribution of corneal astigmatism

Of the 502,642 UKBB participants who had keratometry measures, after exclusions, 83,751 participants were selected for the purpose of this study. Of these, 36,490 (44%) were male. Ethnicity was 90% white, 3.44% Asian, 3.01% black, 0.89% mixed and 0.41% Chinese (Table 1). In the right eye, 69%, 46%, 29%, 11% and 5% had corneal astigmatism greater than or equal to 0.5, 0.75, 1.0, 1.5 and 2.0 dioptres respectively, and in the left eye, 69%, 46%, 30%, 11% and 5% (Fig 1). After stratification of participants by age group (decade) and gender, corneal astigmatism was found to decrease with age and to be on average higher in females than in males across age groups in the UKBB (Table 2). There was a small but significant difference between corneal astigmatism in left and right eye, with left eye corneal astigmatism higher by on average 0.009 dioptres. A difference between left and right eye corneal astigmatism of less than 1 dioptre was found in 95% of individuals and a difference of more than 2 dioptres (anisometropia) was found in the 0.83% of eyes. This left right asymmetry has previously been reported by Cumberland et al (2015) [17], and is likely due to right eyes always being measured before left eyes according to the UKBB protocol for acquiring keratometry measures (https://biobank.ndph.ox.ac.uk/showcase/showcase/docs/Refraction.pdf, Section 5.1).

Association of mean corneal power and axis of astigmatism with age

Older age was significantly associated with increased mean corneal power in both eyes, with an average increase of 0.15 dioptres per decade (Fig C in S1 File). Axis of astigmatism changed with older age from with-the-rule to against-the-rule (Fig G in S1 File) as reported by Yu et al [19].

Table 1. Distribution of participants in the UKBB across the different variables. Mean/sd or percentage of the 83,751 study participants in the UKBB by sex. P values estimated by a t-test for continuous variables and chi-squared test for categorical variables in order to determine whether there is a systematic difference between males and females.

variable	total	males	females	P value
Age, years	57.1 (8.1)	57.3 (8.2)	56.9 (8.0)	< .001
Ethnicity white	92.1	92.2	92	< .001
Ethnicity asian	3.5	4	3.1	
Ethnicity black	3.1	2.8	3.3	
Ethnicity mixed	0.9	0.7	1	
Ethnicity chinese	0.4	0.3	0.5	
Age completed full time education	16.9 (2.5)	16.9 (2.7)	16.8 (2.3)	< .001
Skin colour *	2.3 (0.9)	2.4 (0.9)	2.3 (0.9)	< .001
Use of UV protection	2.7 (0.9)	2.5 (0.9)	2.9 (0.9)	< .001
Alcohol intake **	3.0 (1.6)	3.3 (1.5)	2.8 (1.6)	< .001
Season of assessment spring	34.6	34.7	34.6	
Season of assessment autumn	23.7	23.5	23.8	
Season of assessment winter	22.1	22.4	21.9	
Season of assessment summer	19.6	19.5	19.7	
Corneal-compensated IOP, mmHg	16.1 (4.2)	16.4 (4.2)	15.9 (4.3)	< .001
Corneal resistance factor	10.7 (2.3)	10.6 (2.3)	10.9 (2.4)	< .001
Corneal hysteresis	10.6 (2.3)	10.4 (2.2)	10.8 (2.3)	< .001
Height, m	168.3 (9.2)	175.7 (6.8)	162.7 (6.3)	< .001
Weight, 10 kg	77.4 (15.8)	85.5 (14.2)	71.3 (14.0)	< .001
BMI, kg/m^2	27.3 (4.8)	27.7 (4.2)	27.0 (5.1)	< .001
SBP, mmHg	139.7 (19.5)	142.3 (18.3)	137.6 (20.2)	< .001
DBP, mmHg	81.9 (10.6)	83.6 (10.4)	80.5 (10.5)	< .001
Townsend deprivation index	-1.0 (3.0)	-1.0 (3.0)	-1.0 (2.9)	
Smoker: true	90.1	87.9	91.8	< .001
Smoker: false	9.9	12.1	8.2	
Age Asthma Diagnosed, self-reported	30.5 (18.5)	27.3 (19.1)	32.7 (17.7)	< .001
Diabetes, doctor diagnosed: true	94.8	93.1	96.1	< .001
Diabetes, doctor diagnosed: false	5.2	6.9	3.9	

* Skin colour is coded as: very fair = 1, fair = 2, light olive = 3, dark olive = 4, brown = 5, black = 6

** Alcohol intake is coded as: never = 0, special occasions = 1, one to three times a month = 2, once twice a week = 3, three four times a week = 4, daily = 5.

https://doi.org/10.1371/journal.pone.0218144.t001

Association of amblyopia and strabismus with corneal astigmatism

The number of eyes affected by amblyopia and strabismus in the UKBB are 2483 and 1052 respectively. Corneal astigmatism was highest in eyes affected by amblyopia and strabismus (Fig D in S1 File). This confirms that high corneal astigmatism is a significant risk factor for amblyopia (OR = 1.98 (1.87 to 2.09), P<0.001) and strabismus (OR = 1.73 (1.59 to 1.88), P<0.001) (Fig 4 in S1 File) [20,21].

Univariable and multivariable analysis of corneal astigmatism

Univariable analysis revealed that, by decreasing magnitude of effect size—female gender, white ethnicity, lighter skin colour, use of UV protection, lower alcohol intake, lower ccIOP, older age at completion of education, younger age, higher Townsend deprivation index, lower height and lower systolic blood pressure–were significantly associated with

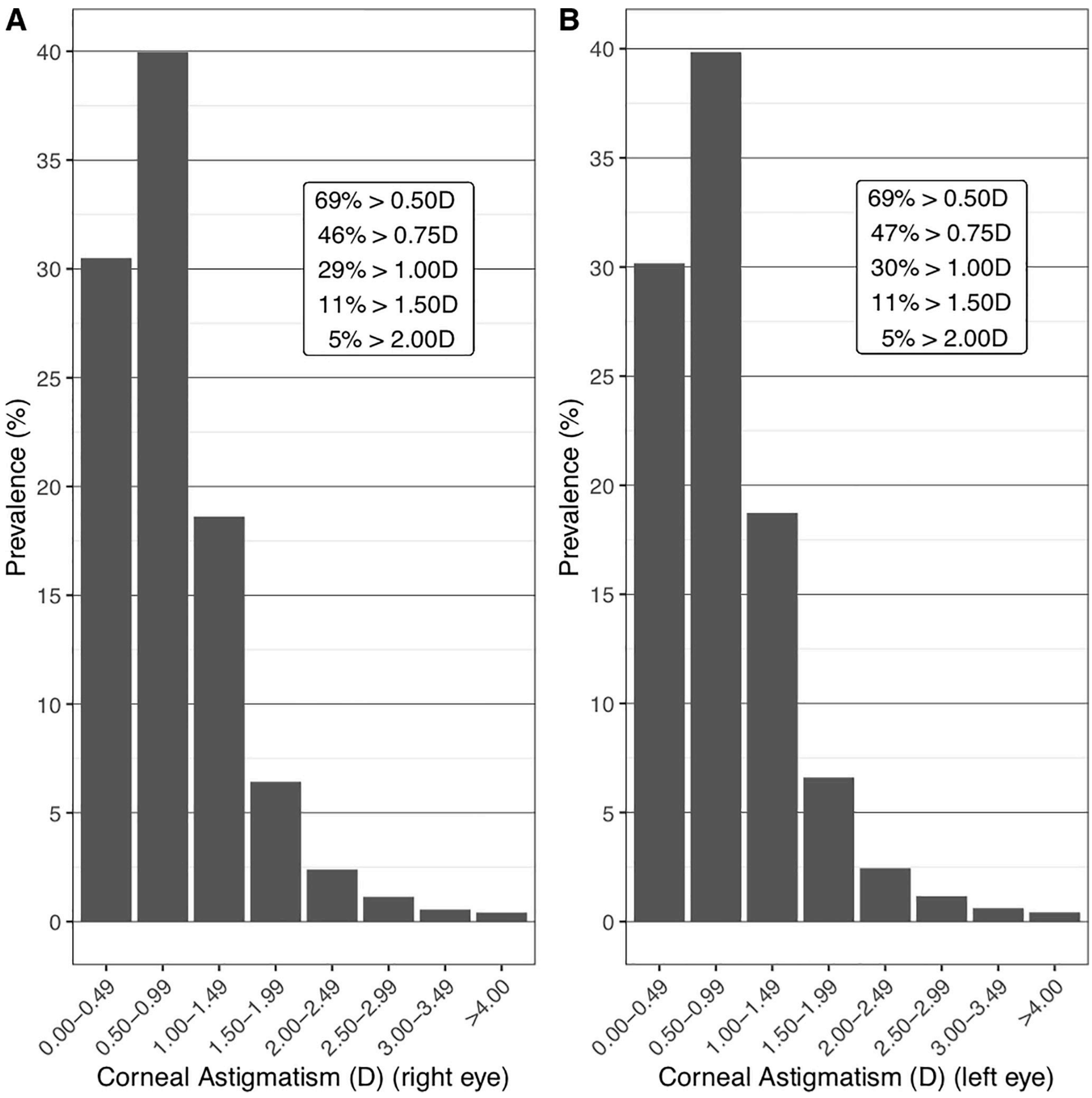

Fig 1. Distribution of corneal astigmatism in the UKBB in bins of 0.5D. (A) Distribution of corneal astigmatism in right eye. (B) Distribution of corneal astigmatism in left eye.

https://doi.org/10.1371/journal.pone.0218144.g001

increased corneal astigmatism (Table 3 and Table B in S1 File). After including these variables in the multivariable analysis (Table 4 and Table C in S1 File), the following parameters remained significantly associated with increased corneal astigmatism: female gender, white ethnicity, lighter skin colour, use of UV protection, lower alcohol intake, lower ccIOP, older age at completion of education, younger age and higher Townsend deprivation index.

Table 2. Mean, standard deviation, 25th and 75th percentile of right and left corneal astigmatism by age and gender of the 83,751 study participants in the UK Biobank. Corneal astigmatism decreases slightly with age and is slightly higher in females than in males. Left corneal astigmatism tends to be very slightly but significantly higher than right corneal astigmatism (P < 0.001). P values derived from a t-test between right and left eye corneal astigmatism across all cohorts.

cohorts	right eye 3mm corneal astigmatism	left eye 3mm corneal astigmatism
Men 40–49	0.861 (0.683, 0.430–1.090)	0.864 (0.674, 0.440–1.100)
Men 50–59	0.810 (0.640, 0.400–1.030)	0.818 (0.646, 0.400–1.050)
Men 60–69	0.788 (0.619, 0.390–1.000)	0.792 (0.627, 0.400–1.000)
Women 40–49	0.923 (0.625, 0.510–1.190)	0.942 (0.638, 0.520–1.200)
Women 50–59	0.890 (0.611, 0.470–1.140)	0.915 (0.645, 0.490–1.170)
Women 60–69	0.850 (0.619, 0.440–1.090)	0.855 (0.619, 0.440–1.090)
Total 40–49	0.896 (0.652, 0.470–1.150)	0.908 (0.655, 0.480–1.160)
Total 50–59	0.857 (0.624, 0.440–1.100)	0.875 (0.647, 0.450–1.120)
Total 60–69	0.822 (0.620, 0.420–1.050)	0.827 (0.623, 0.420–1.050)
All	0.848 (0.627, 0.430–1.090)	0.857 (0.636, 0.440–1.100)
Difference (right-left)		-0.009 (-0.012–0.01), P<0.001

https://doi.org/10.1371/journal.pone.0218144.t002

Discussion

Distribution of corneal astigmatism in the UKBB compared to other cohorts

The distribution of astigmatism in the large population reported in this study supports evidence from previous smaller studies, both in the UK and worldwide, in pre-operative patients [11,12,22–25] and from large consortiums such as CREAM (n = 55,177) [13]. We found that 69%, 29%, 11% and 5% had corneal astigmatism ≥0.5, 1.0, 1.5 and 2.0 dioptres respectively. These are slightly lower than values reported from a recent series of 110,468 cataract pre-operative eyes [25] where 78%, 42%, 21% and 11% having corneal astigmatism ≥0.5, 1.0, 1.5 and 2.0 dioptres respectively. A study of 1,230 eyes undergoing cataract surgery in Wales found corneal astigmatism of >0.5D in 75% in Wales [12] (N = 1,230 eyes). Corneal astigmatism ≥1.0D was found in 36% of eyes with cataract in Germany [26] (N = 15,448 eyes), 47% in China (N = 12,449)[23] and 35% in South Korea [22] (N = 2,847 eyes). Recently, Curragh et al [24] reported that 41% of eyes undergoing cataract surgery (N = 2,080) in Northern Ireland had >1.0D of corneal astigmatism. However, cataract surgery is usually performed in an older age group than those of the participants in the UK Biobank and these pre-operative clinical groups are not necessarily comparable to UKBB participants whose age range is between 40 and 69. A recent CREAM study [27] reported the median corneal astigmatism and median age across 22 studies (8 Asian and 14 European). The median corneal astigmatism was reported in each study and this ranged from 0.539D in the Rotterdam-II European study (N = 3964, mean age = 64.8) [28], to 1.21D in the Asian Singapore Cohort Study of the Risk Factors for Myopia (SCORM) study (N = 1894, mean age = 10.8) [29]. Comparable studies to the UKBB in terms of age and gender demographics of the participants are the Rotterdam-III Study (N = 5850 eyes, mean age = 57)[30], the Singapore Chinese Eye Study (SCES-610K) (N = 1106 eyes, mean age = 57.6)[31], the Gutenberg Health Study (GHS-1) study (N = 4796 eyes, mean age = 55.9)[32] which reported a median corneal astigmatism of 0.618D, 0.703D and 0.65D respectively. This is comparable to the UKBB median corneal astigmatism of 0.71D.

Table 3. Results of univariable regression in 83,751 study participants in the UKBB for right and left eye corneal astigmatism. Significant associations are highlighted in bold. P values for B coefficients are derived from a t-test. 95% CI = 95% confidence interval.

Description	Right eye univariable B (95% CI)	P value	Left eye univariable B (95% CI)	P value
**Age, years**	**-0.004 (-0.004 to -0.003)**	**<0.001**	**-0.004 (-0.005 to -0.004)**	**<0.001**
**Sex (Ref = F)**	**-0.070 (-0.079 to -0.061)**	**<0.001**	**-0.080 (-0.089 to -0.071)**	**<0.001**
**Ethnicity (Ref = white) asian**	**-0.081 (-0.104 to -0.058)**	**<0.001**	**-0.102 (-0.126 to -0.079)**	**<0.001**
**black**	**-0.061 (-0.086 to -0.036)**	**<0.001**	**-0.068 (-0.093 to -0.043)**	**<0.001**
mixed	-0.017 (-0.063 to 0.028)	0.45	-0.018 (-0.064 to 0.028)	0.447
chinese	-0.022 (-0.089 to 0.044)	0.515	-0.040 (-0.108 to 0.027)	0.243
**Age completed full time education**	**0.004 (0.002 to 0.006)**	**0.001**	**0.005 (0.003 to 0.007)**	**<0.001**
**Skin colour, lighter to darker***	**-0.029 (-0.034 to -0.024)**	**<0.001**	**-0.030 (-0.035 to -0.026)**	**<0.001**
**Use of UV protection**	**0.017 (0.013 to 0.022)**	**<0.001**	**0.016 (0.011 to 0.020)**	**<0.001**
**Alcohol intake, never to daily****	**-0.009 (-0.012 to -0.006)**	**<0.001**	**-0.009 (-0.012 to -0.007)**	**<0.001**
Season of assessment (baseline = spring) autumn	-0.005 (-0.016 to 0.006)	0.387	-0.011 (-0.023 to 0.000)	0.055
winter	-0.007 (-0.019 to 0.004)	0.212	-0.006 (-0.018 to 0.006)	0.324
summer	0.017 (0.005 to 0.029)	0.005	0.014 (0.002 to 0.026)	0.026
**Corneal-compensated IOP, mmHg**	**-0.006 (-0.007 to -0.005)**	**<0.001**	**-0.007 (-0.008 to -0.006)**	**<0.001**
Corneal resistance factor	-0.003 (-0.005 to -0.002)	<0.001	-0.001 (-0.003 to 0.001)	0.35
Corneal hysteresis	0.003 (0.001 to 0.005)	0.004	0.006 (0.004 to 0.007)	<0.001
**Height, m**	**-0.002 (-0.002 to -0.002)**	**<0.001**	**-0.002 (-0.002 to -0.001)**	**<0.001**
Weight, 10 kg	-0.000 (-0.001 to -0.000)	0.012	-0.000 (-0.001 to -0.000)	0.044
BMI, kg/m^2	0.001 (0.000 to 0.002)	0.021	0.001 (0.000 to 0.002)	0.011
**SBP, mmHg**	**-0.001 (-0.001 to -0.001)**	**<0.001**	**-0.001 (-0.001 to -0.001)**	**<0.001**
DBP, mmHg	-0.001 (-0.001 to -0.000)	0.001	-0.001 (-0.001 to -0.000)	<0.001
**Townsend deprivation index**	**0.003 (0.001 to 0.004)**	**<0.001**	**0.003 (0.002 to 0.005)**	**<0.001**
Smoker (baseline = false)	-0.011 (-0.025 to 0.004)	0.144	-0.012 (-0.026 to 0.003)	0.112
Age Asthma Diagnosed, self-reported	-0.001 (-0.002 to -0.000)	0.014	-0.001 (-0.002 to -0.000)	0.014
Diabetes, doctor diagnosed (baseline = false)	-0.023 (-0.042 to -0.004)	0.018	-0.006 (-0.025 to 0.014)	0.561

* Skin colour is coded as: very fair = 1, fair = 2, light olive = 3, dark olive = 4, brown = 5, black = 6

** Alcohol intake is coded as: never = 0, special occasions = 1, one to three times a month = 2, once twice a week = 3, three four times a week = 4, daily = 5.

https://doi.org/10.1371/journal.pone.0218144.t003

Modelling of corneal astigmatism

In the multivariable analysis, parameters known to be strongly associated with gender, such as height and weight (Table 4), were no longer significantly associated with corneal astigmatism. Variables which remained significantly associated by decreasing order of magnitude were gender, skin colour, alcohol intake, age at completion of full-time education, ccIOP, age and Townsend deprivation score. The adjusted R-squared of the multivariable regression was remarkably low at 0.01 which highlights that there are many other unobserved variables which influence corneal astigmatism.

Gender. Our study confirms, as previously reported by Yuan et al [23], that corneal astigmatism is higher in females than in males even after adjusting for weight and height (Table 4). Females have on average 0.07D more corneal astigmatism in right eye and 0.08D more in left eye (Table 3).

Ethnicity and skin colour. Asian and black ethnicities appear to be significantly protective for corneal astigmatism in both eyes according to the univariable analysis (Table 3 and

Table 4. Results of multivariable regression in 83,751 study participants in the UKBB for right and left eye corneal astigmatism. Only parameters that were significant in the univariable regression were included in the multivariable regression. Significant associations are highlighted in bold. P values for B coefficients are derived from a t-test. 95% CI = 95% confidence interval.

Description	Right eye multivariable B (95% CI)	P value	Left eye multivariable B (95% CI)	P value
**Age, years**	**-0.004 (-0.004 to -0.003)**	**<0.001**	**-0.004 (-0.004 to -0.003)**	**<0.001**
**Sex (Ref = F)**	**-0.053 (-0.070 to -0.037)**	**<0.001**	**-0.079 (-0.095 to -0.062)**	**<0.001**
Ethnicity (Ref = white) asian	-0.026 (-0.065 to 0.014)	0.208	-0.072 (-0.113 to -0.032)	<0.001
black	0.015 (-0.030 to 0.061)	0.508	-0.018 (-0.064 to 0.028)	0.448
mixed	-0.064 (-0.126 to -0.002)	0.044	-0.032 (-0.095 to 0.032)	0.327
chinese	-0.088 (-0.188 to 0.012)	0.085	-0.079 (-0.181 to 0.023)	0.127
**Age completed full time education**	**0.005 (0.002 to 0.007)**	**<0.001**	**0.005 (0.003 to 0.008)**	**<0.001**
**Skin colour, lighter to darker***	**-0.033 (-0.042 to -0.024)**	**<0.001**	**-0.028 (-0.037 to -0.019)**	**<0.001**
Use of UV protection	0.007 (0.001 to 0.013)	0.031	0.000 (-0.006 to 0.007)	0.901
**Alcohol intake, never to daily****	**-0.008 (-0.012 to -0.004)**	**<0.001**	**-0.007 (-0.011 to -0.003)**	**<0.001**
**Corneal-compensated IOP, mmHg**	**-0.005 (-0.006 to -0.004)**	**<0.001**	**-0.006 (-0.008 to -0.005)**	**<0.001**
Corneal resistance factor	-0.006 (-0.008 to -0.003)	<0.001		
Corneal hysteresis			-0.003 (-0.005 to -0.000)	0.037
Height, m	-0.001 (-0.002 to -0.000)	0.042	-0.000 (-0.001 to 0.001)	0.742
SBP, mmHg	-0.000 (-0.000 to 0.000)	0.702	-0.000 (-0.001 to 0.000)	0.258
DBP, mmHg	0.001 (0.000 to 0.002)	0.021	0.001 (0.000 to 0.001)	0.038
**Townsend deprivation index**	**0.004 (0.002 to 0.006)**	**<0.001**	**0.004 (0.002 to 0.006)**	**<0.001**

* Skin colour is coded as: very fair = 1, fair = 2, light olive = 3, dark olive = 4, brown = 5, black = 6

** Alcohol intake is coded as: never = 0, special occasions = 1, one to three times a month = 2, once twice a week = 3, three four times a week = 4, daily = 5.

https://doi.org/10.1371/journal.pone.0218144.t004

Table B in S1 File) but are no longer significant in the multivariable analysis (Table 4 and Table 3 in S1 File). However, skin colour remains significantly associated with darker skin being protective (B = -0.032 (-0.042 to -0.024), P<0.001) (Table 4 and Table C in S1 File). This relationship can also be clearly seen independently in males and females (Fig E in S1 File). The link between corneal astigmatism and deficiency in melanin production has been previously reported for albinism [33]. Our data suggests that darker skin and hence possibly increased melanin production appears protective for corneal astigmatism.

Alcohol intake. Alcohol intake is significantly protective for corneal astigmatism according to the univariable and multivariable analysis (B = -0.008, (-0.012 to -0.004), P<0.001). In particular, the group that drinks nearly every day has the lowest average corneal astigmatism at 0.80 dioptres. This is surprising due to the negative consequences of alcohol abuse on eye conditions. However, on closer inspection it appears that the group that drinks nearly every day in the UKBB consists primarily of men in the 65+ age group; 55% of men drink every day in this study vs 44% of women. Alcohol intake effect is difficult to decouple from gender and age due to the three-way interaction between alcohol-intake, age and gender, with "never-drinkers" and "daily drinkers" showing a clear interaction (Fig F in S1 File).

Age completed full-time education. We found a significant positive association between age at which full-time education was completed and corneal astigmatism (B = 0.006 (0.004 to 0.008), P<0.001). As far as we know, this association has not been reported before. This result was consistent with participants with self-reported astigmatism finishing full-time education later than other participants (Fig D in S1 File). Interestingly, this relationship was not observed in individuals with myopia (Fig D in S1 File), which may be supported by recent evidence

suggesting that myopia is not linked as much to near work [34], but rather to earlier life exposures [35].

Hysteresis and corneal-compensated intraocular pressure. Of interest, corneal hysteresis, which measures the cornea's ability to absorb and dissipate energy, was not found to be associated with corneal astigmatism in the univariable nor in the multivariable analysis.

In the univariable analysis (Table 3 and Table B in S1 File), we found a small but significant protective effect of ccIOP on corneal astigmatism in both eyes (B = -0.006 (-0.007 to -0.005), P<0.001), which remained significant in the multivariable analysis (B = -0.005 (-0.006 to -0.004), P<0.001) (Table 4 and Table C in S1 File). As far as we know, the significant association of ccIOP with corneal astigmatism has not been detected before although it has been tested for in a small study [36].

Age. In the age range of the UKBB, from age 40 to 69, corneal astigmatism decreased significantly with age by an average of 0.04 dioptres in both eyes per decade in the univariable and multivariable analysis (Table 4). This result is supported by Yuan et al [23]. However, in a previous UKBB study, Shah et al [37] reported that the level of corneal astigmatism is relatively constant across age groups. However, no linear regression was performed by the authors to support this statement. They did find however that increasing age is associated with higher refractive astigmatism, as derived from cylindrical power, which we have also recapitulated (B = 0.015 (0.014 to 0.016), P<0.001) (Fig C in S1 File). We believe this increase in refractive astigmatism with age is likely driven by increasing lenticular astigmatism, since corneal astigmatism decreases.

Townsend deprivation index. Higher corneal astigmatism is associated with a higher Townsend deprivation index (B = 0.004 (0.002 to 0.006), P<0.001) in the UKBB (Table 4). To our knowledge this association has never been reported before. This association may be partly due to an interaction effect with age since older individuals, hence with lower corneal astigmatism, tend to live in less deprived areas (Fig H in S1 File).

Strengths and limitations of our study

The strength of this study is the large sample size of 83,751 participants and that participants were not pre-operative patients hence more representative of the general population. However due to the limited age range of the participants, between 40 and 69 years, the age distribution is limited to adult life and are results cannot be extrapolated outside that age range. For instance, we cannot confirm whether or not corneal astigmatism increases past the age of 70 [19,23] as none of the UKBB participants had refractometry past the age of 69. Furthermore, due to the voluntary nature of the UKBB study, participants are likely to be a healthier more educated sample of the UK population and not necessarily representative of the general UK population. Regardless, a range of exposures and characteristics are likely to have been captured due to the sample size of the UKBB and so the results can still be applicable to other populations.

Conclusion

This analysis confirms, in a dataset of 83,751 individuals within the 40–69 age range, adverse associations with corneal astigmatism such as younger age and female gender, and identified novel associations including lighter skin colour, lower frequency of alcohol intake, later age having completed full time education, lower ccIOP and higher Townsend deprivation index. Further research and longitudinal data are needed to investigate these novel associations and to infer causality.

Supporting information

S1 File. Supplementary materials.
(DOCX)

Acknowledgments

This research has been conducted using the UK Biobank Resource under Application Number 10536. Collaborators on the application are Nikolas Pontikos, Alexander Day, Parul Desai, Paul Foster and Stephen Tuft. The PI is Alexander Day. The main contact for this consortium is Prof Paul Foster (p.foster@ucl.ac.uk) and list of members is available from the consortium website (http://www.ukbiobankeyeconsortium.org.uk/people) and below.

Disclaimer

The views expressed are those of the authors and not necessarily those of the NHS, the NIHR or the Department of Health.

Collaborators on behalf of the UK Biobank

UK Biobank Eye & Vision Consortium: The UK Biobank Eye & Vision Consortium members are Tariq Aslam, PhD, Manchester University, Sarah A. Barman, PhD, Kingston University, Jenny H. Barrett, PhD, University of Leeds, Paul Bishop, PhD, Manchester University, Peter Blows, BSc, NIHR Biomedical Research Centre, Catey Bunce, DSc, King's College London, Roxana O. Carare, PhD, University of Southampton, Usha Chakravarthy, FRCOphth, Queens University Belfast, Michelle Chan, FRCOphth, NIHR Biomedical Research Centre, Sharon Y. L. Chua, PhD, NIHR Biomedical Research Centre, David P. Crabb, PhD, UCL, Philippa M. Cumberland, MSc, UCL Great Ormond Street Institute of Child Health, Alexander Day, PhD, NIHR Biomedical Research Centre, Parul Desai, PhD, NIHR Biomedical Research Centre, Bal Dhillon, FRC Ophth, University of Edinburgh, Andrew D. Dick, FRC Ophth, University of Bristol, Cathy Egan, FRC Ophth, NIHR Biomedical Research Centre, Sarah Ennis, PhD, University of Southampton, Paul Foster, PhD, NIHR Biomedical Research Centre, Marcus Fruttiger, PhD, NIHR Biomedical Research Centre, John E.J. Gallacher, PhD, University of Oxford, David F. Garway-Heath MD FRCOphth—NIHR Biomedical Research Centre, Jane Gibson, PhD, University of Southampton, Dan Gore, FRCOphth, NIHR Biomedical Research Centre, Jeremy A. Guggenheim, PhD, Cardiff University, Chris J. Hammond, FRCOphth, King's College London, Alison Hardcastle, PhD, NIHR Biomedical Research Centre, Simon P. Harding, MD, University of Liverpool, Ruth E. Hogg, PhD, Queens University Belfast, Pirro Hysi, PhD, King's College London, Pearse A. Keane, MD, NIHR, Biomedical Research Centre, Sir Peng T. Khaw, PhD, NIHR Biomedical Research Centre, Anthony P. Khawaja, DPhil, NIHR Biomedical Research Centre, Gerassimos Lascaratos, PhD, NIHR Biomedical Research Centre, Andrew J. Lotery, MD, University of Southampton, Tom Mac Gillivray, PhD, University of Edinburgh, Sarah Mackie, PhD, University of Leeds, Keith Martin, FRCOphth, University of Cambridge, Michelle Mc Gaughey, Queen's University Belfast, Bernadette McGuinness, PhD, Queen's University Belfast, Gareth J. McKay, PhD, Queen's University Belfast, Martin McKibbin, FRC Ophth, Leeds Teaching Hospitals NHS Trust, Danny Mitry, PhD, NIHR Biomedical Research Centre, Tony Moore, FRCOphth, NIHR Biomedical Research Centre, James E. Morgan, DPhil, Cardiff University, Zaynah A. Muthy, BSc, NIHR Biomedical Research Centre, Eoin O'Sullivan, MD, King's College Hospital NHS Foundation Trust, Chris G. Owen, PhD, University of London, Praveen Patel, FRCOphth, NIHR Biomedical Research Centre, Euan Paterson, BSc, Queens University Belfast, Tunde Peto, PhD, Queen's University Belfast, Axel

Petzold, PhD, UCL, Jugnoo S. Rahi, PhD, UCL Great Ormond Street Institute of Child Health, Alicja R. Rudnikca, PhD, University of London, Jay Self, PhD, University of Southampton, Sobha Sivaprasad, FRC Ophth, NIHR Biomedical Research Centre, David Steel, FRCOphth, Newcastle University, Irene Stratton, MSc, Gloucestershire Hospitals NHS Foundation Trust, Nicholas Strouthidis, PhD, NIHR Biomedical Research Centre, Cathie Sudlow, DPhil, University of Edinburgh, Dhanes Thomas, FRC Ophth, NIHR Biomedical Research Centre, Emanuele Trucco, PhD, University of Dundee, Adnan Tufail, FRCOphth, NIHR Biomedical Research Centre, Veronique Vitart, PhD, University of Edinburgh, Stephen A. Vernon, DM, Nottingham University Hospitals NHS Trust, Ananth C. Viswanathan, FRCOphth, NIHR Biomedical Research Centre, Cathy Williams, PhD, University of Bristol, Katie Williams, PhD, King's College London, Jayne V. Woodside, MRC Ophth, PhD, Queen's University Belfast, Max M. Yates, PhD, University of East Anglia, Jennifer Yip, PhD, University of Cambridge, and Yalin Zheng, PhD, University of Liverpool.

Author Contributions

Conceptualization: Nikolas Pontikos, Stephen J. Tuft, Alexander C. Day.

Data curation: Nikolas Pontikos.

Formal analysis: Nikolas Pontikos.

Funding acquisition: Nikolas Pontikos, Stephen J. Tuft, Alexander C. Day.

Investigation: Nikolas Pontikos.

Methodology: Nikolas Pontikos.

Resources: Nikolas Pontikos, Stephen J. Tuft.

Software: Nikolas Pontikos.

Supervision: Stephen J. Tuft, Alexander C. Day.

Validation: Nikolas Pontikos.

Visualization: Nikolas Pontikos.

Writing – original draft: Nikolas Pontikos, Stephen J. Tuft, Alexander C. Day.

Writing – review & editing: Nikolas Pontikos, Sharon Chua, Paul J. Foster, Stephen J. Tuft, Alexander C. Day.

References

1. Bourne RRA, Stevens GA, White RA, Smith JL, Flaxman SR, Price H, et al. Causes of vision loss worldwide, 1990–2010: A systematic analysis. Lancet Glob Heal. 2013;
2. Hashemi H, Fotouhi A, Yekta A, Pakzad R, Ostadimoghaddam H, Khabazkhoob M. Global and regional estimates of prevalence of refractive errors: Systematic review and meta-analysis. Journal of Current Ophthalmology. 2018.
3. Morlet N, Minassian D, Dart J. Astigmatism and the analysis of its surgical correction. The British journal of ophthalmology. 2002.
4. Hirsch MJ. Changes in astigmatism after the age of forty. Optom Vis Sci. 1959;
5. Attebo K, Ivers RQ, Mitchell P. Refractive errors in an older population: The Blue Mountains Eye Study. Ophthalmology. 1999;
6. Hayashi K, Hayashi H, Hayashi F. Topographic Analysis of the Changes in Corneal Shape Due to Aging. Cornea. 1995;
7. Riley AF, Grupcheva CN, Malik TY, Craig JP, McGhee CNJ. The Auckland Cataract Study: Demographic, corneal topographic and ocular biometric parameters. Clin Exp Ophthalmol. 2001;

8. Ho JD, Liou SW, Tsai RJF, Tsai CY. Effects of aging on anterior and posterior corneal astigmatism. Cornea. 2010;
9. Mohammadi M, Naderan M, Pahlevani R, Jahanrad A. Prevalence of corneal astigmatism before cataract surgery. Int Ophthalmol. 2016;
10. Hoffer KJ. Biometry of 7,500 cataractous eyes. Am J Ophthalmol. 1980;
11. Hoffmann PC, Hütz WW. Analysis of biometry and prevalence data for corneal astigmatism in 23 239 eyes. J Cataract Refract Surg. 2010;
12. Khan MI, Muhtaseb M. Prevalence of corneal astigmatism in patients having routine cataract surgery at a teaching hospital in the United Kingdom. J Cataract Refract Surg. 2011;
13. Verhoeven VJM, Hysi PG, Saw SM, Vitart V, Mirshahi A, Guggenheim JA, et al. Large scale international replication and meta-analysis study confirms association of the 15q14 locus with myopia. The CREAM consortium. Hum Genet. 2012;
14. Ollier W, Sprosen T, Peakman T. UK Biobank: From concept to reality. Pharmacogenomics. 2005;
15. Palmer LJ. UK Biobank: bank on it. Lancet. 2007.
16. Allen N, Sudlow C, Downey P, Peakman T, Danesh J, Elliott P, et al. UK Biobank: Current status and what it means for epidemiology. Heal Policy Technol. 2012;
17. Cumberland PM, Bao Y, Hysi PG, Foster PJ, Hammond CJ, Rahi JS. Frequency and distribution of refractive error in adult life: Methodology and findings of the UK biobank study. PLoS One. 2015;
18. Ueno Y, Hiraoka T, Beheregaray S, Miyazaki M, Ito M, Oshika T. Age-Related Changes in Anterior, Posterior, and Total Corneal Astigmatism. J Refract Surg. 2014;
19. Yu JG, Zhong J, Mei ZM, Zhao F, Tao N, Xiang Y. Evaluation of biometry and corneal astigmatism in cataract surgery patients from Central China. BMC Ophthalmol. 2017;
20. Jonas JB, Kling F, Grundler AE. Optic disc shape, corneal astigmatism, and amblyopia. Ophthalmology. 1997;
21. Harvey EM. Development and treatment of astigmatism-related amblyopia. Optometry and Vision Science. 2009.
22. Oh EH, Kim H, Lee HS, Hwang KY, Joo CK. Analysis of anterior corneal astigmatism before cataract surgery using power vector analysis in eyes of Korean patients Presented in part at the 110th annual meeting of the Korean Ophthalmological Society, Seoul, South Korea, November 2013. J Cataract Refract Surg [Internet]. 2015 Jun [cited 2019 Aug 11]; 41(6):1256–63. Available from: http://www.ncbi.nlm.nih.gov/pubmed/26189381 https://doi.org/10.1016/j.jcrs.2014.09.043 PMID: 26189381
23. Yuan X, Song H, Peng G, Hua X, Tang X. Prevalence of corneal astigmatism in patients before cataract surgery in Northern China. J Ophthalmol. 2014;
24. Curragh DS, Hassett P. Prevalence of corneal astigmatism in an NHS cataract surgery practice in Northern Ireland. Ulster Med J. 2017;
25. Day AC, Dhariwal M, Keith MS, Ender F, Perez Vives C, Miglio C, et al. Distribution of preoperative and postoperative astigmatism in a large population of patients undergoing cataract surgery in the UK. Br J Ophthalmol. 2019;
26. Schuster AKG, Pfeiffer N, Schulz A, Hoehn R, Ponto KA, Wild PS, et al. Refractive, corneal and ocular residual astigmatism: distribution in a German population and age-dependency—the Gutenberg health study. Graefe's Arch Clin Exp Ophthalmol. 2017;
27. Shah RL, Li Q, Zhao W, Tedja MS, Tideman JWL, Khawaja AP, et al. A genome-wide association study of corneal astigmatism: The CREAM Consortium. Mol Vis. 2018;
28. Hofman A, Breteler MMB, Van Duijn CM, Krestin GP, Pols HA, Stricker BHC, et al. The Rotterdam Study: Objectives and design update. Eur J Epidemiol. 2007;
29. Saw SM, Shankar A, Tan SB, Taylor H, Tan DTH, Stone RA, et al. A cohort study of incident myopia in Singaporean children. Investig Ophthalmol Vis Sci. 2006;
30. Hofman A, Brusselle GGO, Murad SD, van Duijn CM, Franco OH, Goedegebure A, et al. The Rotterdam Study: 2016 objectives and design update. Eur J Epidemiol. 2015;
31. R. L, V.S.E. J, Y. Z, P. R, N. C, E.S. T, et al. Methodology of the Singapore Indian Chinese Cohort (SICC) eye study: Quantifying ethnic variations in the epidemiology of eye diseases in Asians. Ophthalmic Epidemiol [Internet]. 2009; 16(6):325–36. Available from: http://ovidsp.ovid.com/ovidweb.cgi?T=JS&PAGE=reference&D=emed12&NEWS=N&AN=355791759 https://doi.org/10.3109/09286580903144738 PMID: 19995197
32. Höhn R, Kottler U, Peto T, Blettner M, Münzel T, Blankenberg S, et al. The ophthalmic branch of the Gutenberg health study: Study design, cohort profile and self-reported diseases. PLoS One. 2015;

33. Schulze Schwering M, Kumar N, Bohrmann D, Msukwa G, Kalua K, Kayange P, et al. Refractive errors, visual impairment, and the use of low-vision devices in albinism in Malawi. Graefe's Arch Clin Exp Ophthalmol. 2015;
34. Cooper J, Tkatchenko A V. A Review of Current Concepts of the Etiology and Treatment of Myopia. Eye Contact Lens. 2018;
35. Tedja MS, Wojciechowski R, Hysi PG, Eriksson N, Furlotte NA, Verhoeven VJM, et al. Genome-wide association meta-analysis highlights light-induced signaling as a driver for refractive error. Nat Genet. 2018;
36. Hagishima M, Kamiya K, Fujimura F, Morita T, Shoji N, Shimizu K. Effect of corneal astigmatism on intraocular pressure measurement using ocular response analyzer and Goldmann applanation tonometer. Graefe's Arch Clin Exp Ophthalmol. 2010;
37. Shah RL, Guggenheim JA. Genome-wide association studies for corneal and refractive astigmatism in UK Biobank demonstrate a shared role for myopia susceptibility loci. Hum Genet. 2018
